# Quantitating morphological changes in biological samples during scanning electron microscopy sample preparation with correlative super-resolution microscopy

**DOI:** 10.1371/journal.pone.0176839

**Published:** 2017-05-31

**Authors:** Ying Zhang, Tao Huang, Danielle M. Jorgens, Andrew Nickerson, Li-Jung Lin, Joshua Pelz, Joe W. Gray, Claudia S. López, Xiaolin Nan

**Affiliations:** 1 Department of Biomedical Engineering, Knight Cancer Institute, and OHSU Center for Spatial Systems Biomedicine (OCSSB), Oregon Health and Science University, Portland, Oregon, United States of America; 2 Multiscale Microscopy Core, OHSU Center for Spatial Systems Biomedicine, Oregon Health and Science University, Portland, Oregon, United States of America; Pennsylvania State Hershey College of Medicine, UNITED STATES

## Abstract

Sample preparation is critical to biological electron microscopy (EM), and there have been continuous efforts on optimizing the procedures to best preserve structures of interest in the sample. However, a quantitative characterization of the morphological changes associated with each step in EM sample preparation is currently lacking. Using correlative EM and superresolution microscopy (SRM), we have examined the effects of different drying methods as well as osmium tetroxide (OsO_4_) post-fixation on cell morphology during scanning electron microscopy (SEM) sample preparation. Here, SRM images of the sample acquired under hydrated conditions were used as a baseline for evaluating morphological changes as the sample went through SEM sample processing. We found that both chemical drying and critical point drying lead to a mild cellular boundary retraction of ~60 nm. Post-fixation by OsO_4_ causes at least 40 nm additional boundary retraction. We also found that coating coverslips with adhesion molecules such as fibronectin prior to cell plating helps reduce cell distortion from OsO_4_ post-fixation. These quantitative measurements offer useful information for identifying causes of cell distortions in SEM sample preparation and improving current procedures.

## Introduction

Scanning electron microscopy (SEM) is extensively used to study structural details on the surface of biological samples. The conventional sample preparation process for SEM includes fixation, dehydration, drying, and optionally, conductive coating. Fixation is typically performed in aldehyde buffer; in certain cases, this is followed by a post-fixation step in osmium tetroxide (OsO_4_) or uranyl acetate (UA). After fixation, the sample is first dehydrated with organic solvents to replace water and then dried to remove the organic solvents. Each of these steps, i.e., fixation, dehydration, and drying, can introduce artifacts into delicate biological samples such as change in protein localization [1]. Morphological changes were also reported during fixation and drying steps [2]. Much effort has been put into optimizing these procedures to reduce sample preparation artifacts and preserve cell structures and morphology as closely to the native state as possible [3–7]. Such efforts, however, are often based on empirical assessment of the sample quality after preparation, and a quantitative characterization of the morphological changes caused by each step is currently lacking.

It is commonly believed the majority of morphological changes occur in the drying step. Critical point drying (CPD) and chemical drying are most commonly used in SEM sample preparation. In CPD, liquid CO_2_ is added to the sample to replace the organic solvent and then brought to the critical point with increased temperature and pressure, when the liquid and gaseous phases coexist without a boundary. Next, all the liquid is driven to the gas phase by decreasing pressure; this allows removal of liquid from cells without surface tension effects [8]. In chemical drying, an organic solvent is gradually replaced with a volatile chemical with low surface tension, such as hexamethyldisilazane (HMDS), which is then air-dried to completion [9]. HMDS is typically used as a time-saving and cheaper alternative to CPD. In terms of sample preservation, CPD usually is better although some have reported that CPD and HMDS yield similar results [10]. While CPD and HMDS seem to suffice for most biological specimens, drying artifacts such as lines and ridges on the cell surface due to cell shrinkage and even cellular collapse have been documented for both methods [11].

Post-fixation with OsO_4_ is reported to help preserve cellular structure by reacting with lipids, which are the main components of the cell membrane and intracellular organelles but are not fixed by aldehydes. However, OsO_4_ treatment has also been shown to alter cell morphology. For example, Nordestgaard and Rostgaard [12,13] quantitatively traced volume changes by Nomarski differential interference contrast microscopy in isolated hepatocytes during EM specimen preparation. Swelling ranged from 9 to 19% during secondary fixation in 2% OsO_4_. Additionally, OsO_4_ is a strong oxidizing reagent and may cause undesired destruction of membrane components [14], which may be a concern in certain applications such as immuno SEM.

Continued improvement of SEM sample preparation requires a clear understanding of the changes to the specimen during each step, which necessitates the use of light microscopy. Several methods had been used for tracing the volume changes of tissues or cells during each stage of sample preparation. Time-lapse cinematography with light microscopy was used by Boyde [6] and Arborgh [7] to record continuous dimensional changes in cultured cells. Other optical methods including Nomarski [15,16] or phase-contrast optics [4] were also used. Sample shrinkage was also estimated by measuring the gross dimensions of tissue sample [17] or measuring the gross weight loss [18]. However, these methods fall short in distinguishing the influence of each sample preparation step on individual cells. Often times evaluation of sample quality at fine scales has to be based on SEM imaging results when all steps have already been completed, making it difficult to account for the contributions of individual steps.

Recently, the resolution gap between light and electron microscopy has been filled by super-resolution microscopy (SRM) techniques such as photoactivated localization microscopy (PALM) and stochastic optical reconstruction microscopy (STORM). Based on stochastic switching and subdiffractive localization of individual fluorescent molecules, both PALM and STORM offer spatial resolutions on the order of 10 nm. For their high resolving power and compatibility with existing fluorescence labeling approaches, PALM and STORM have been widely used for imaging biological samples to reveal otherwise hidden details [19,20]. More recently, correlative SRM and EM have combined their complementary capabilities in biological imaging, where SRM provides the target specific contrast and EM the morphological features.

In this study, we used SRM to examine morphological changes to the sample during SEM sample preparation. Using SRM images of fully hydrated samples as a standard, we were able to quantitate differences in the cell morphology as the sample goes through different SEM processing steps and imaged with the SEM, after which the two images were precisely registered and compared. We introduced a distortion index as a quantitative indicator for the extent of distortions in cellular shape. Using this approach, we found that the dehydration and drying steps caused a mild boundary retraction at an average of 60 nm to cells cultured on glass, and the effect was similar between CPD and chemical drying using HMDS. Post-fixation by OsO_4_ causes at least an additional 40 nm boundary retraction; this result contrasts current beliefs that OsO_4_ helps to preserve cell morphology. Lastly, we showed that fibronectin coating of coverslips prior to cell plating helps to significantly reduce cell distortion from OsO_4_ post-fixation. These findings offer quantitative insight into factors that affect SEM sample quality, which will be helpful to improve the current procedures for better SEM sample preservation.

## Materials and methods

### Molecular cloning and establishment of stable cell lines

We used two genetically encoded photoactivatable fluorescent proteins, mEos4 and PAmCherry1, to label tubulin and the C-Terminal tail of H-Ras (tH) respectively. The mEos4 plasmid was a gift from Dr. Loren Looger (Janelia Farm)[21]. To generate expression plasmids for mEos4-tubulin and PAmCherry1-tH, PCR fragments used for In-Fusion reactions were generated using Phusion High-Fidelity DNA Polymerase (M0530, New England Biolabs). We used the In-Fusion PCR HD Cloning kit (Catalog number 639649, Clontech) to generate genetic fusions in the pENTR (Life Technologies) backbones, and the Gateway LR Clonase II kit (11791, Life Technologies) to shuttle the resulting fusion constructs from the entry clones to expression clones. Lentiviral backbone (pLenti-puro-CMV/TetOn, 17293, Addgene) was used for expressing these clones. Viral particles containing the pLenti-puro-CMV/Teton-mEos4-tubulin or pLenti-puro-CMV/Teton-PAmCherry1-tH were generated using the ViraPower lentiviral packaging system (K497500, Life Technologies), which were then used to infect U2OS cells (HTB-96, ATCC) to make stable cell lines expressing mEos4-tubulin or PAmCherry1-tH under tetracycline or doxycline regulation. Single clones were isolated, grown out and assayed for repression of Dox-induced gene expression; good clones were used for subsequent studies.

### Sample preparation for correlative microscopy

Cells were cultured at 37°C and 5% CO_2_ in DMEM supplemented with 10% FBS (11995 and 10082 respectively, Life Technologies) and were plated on #1.5 indium-tin-oxide (ITO) coated coverslips (SPI supplies 06486-AB). To grow cells on fibronectin coated coverslips, a 5 or 10 μg/mL fibronectin solution was added to the ITO coated coverslips and incubated at room temperature for 3 hours. Excess fibronectin was removed by aspiration before plating cells. Primary fixation of cells was performed with freshly made 3.0% glutaraldehyde or 3.7% PFA and 0.1% glutaraldehyde in PBS for 20 min at room temperature. For PALM imaging of microtubules, cells were first extracted for 60 seconds with 0.1% Triton X-100 before fixation.

After PALM imaging, the PAmCherry1-tH samples were post-fixed at room temperature with 1%, 2% (v/v) or without OsO_4_. The samples were then dehydrated by a graded series of ethanol (50%, 60%, 70%, 80%, 90%, 95% and three times 100%) at 20 minute intervals. Following dehydration, solvent was removed by either CPD using a critical point dryer (CPD300, Leica) or air drying with HMDS (440191, Sigma-Aldrich). For HMDS drying, the dehydrated specimens were immersed with HMDS for 20 minutes. Then HMDS was decanted, and the samples were left under a hood to air-dry at room temperature. Samples were then coated with 5 nm carbon using a Leica ACE600 Coater before SEM imaging.

To label U2OS cells with wheat germ agglutinin (WGA) for STORM imaging, the cells were first fixed with 3.7% PFA and 0.1% glutaraldehyde. After washing with PBS, the cells were blocked with 3% (w/v) bovine serum albumin (BSA, 9048-46-8, OmniPur) in PBS for 30 minutes. The cells were then incubated with Alexa Fluor 647 conjugated WGA (W32466, Thermo Fisher Scientific) at 1 μg/mL for 30 min before and after OsO_4_ post-fixation. The cells were thoroughly washed with PBS and stored in the same buffer until STORM imaging.

### SRM and SEM imaging and image registration

For imaging mEos4-tubulin and PAmCherry1-tH with PALM, PBS with Ca^2+^/Mg^2+^ (14040133, Life Technologies) was used as the imaging buffer. For imaging WGA-Alexa Fluor 647 with STORM, we used a standard STORM imaging buffer containing 10 mM mercaptoethylamine (MEA, M9768, Sigma-Aldrich) and an oxygen scavenger system [0.5 mg/mL glucose oxidase (G2133, Sigma-Aldrich), 40 μg/mL catalase (C100-50MG, Sigma-Aldrich), and 10% w/v glucose] in a Tris buffer (50 mM Tris supplemented with 20 mM MgCl_2_ and 10 mM NaCl, pH 8). The STORM imaging buffer was made fresh prior to each imaging session.

SRM (i.e., PALM and STORM) imaging was performed on a Nikon Ti-U inverted microscope equipped with a Nikon 60× APO TIRF objective (NA = 1.49). Total internal reflection (TIR) illumination was used in all SRM imaging experiments. Gold particles (~100 nm in size, Cytodiagnotics, G-100-20) were added to the imaging buffer about 20–30 min prior to imaging so they could adhere to the coverslip and be used as fiduciaries. Unlike PALM and STORM fluorophores (e.g. mEos4, PAmCherry1, and Alexa Fluo 647) that undergo photoswitching between frames, these gold nanoparticles emit fluorescence continuously; a stably adherent gold nanoparticle can be localized to at least 10–20 nm precision in each frame and thus can be used as a reference to correct for stage drift during PALM/STORM image acquisition. By averaging trajectories of multiple gold nanoparticles, stage drift could usually be corrected to better than 5 nm. An open source software, μManager[22], was used to acquire raw SRM images. SRM image reconstruction was performed using home-written scripts in Matlab (MathWorks, MA).

SEM images were taken at 2 kV with a FEI Helios 650 Nanolab FIB/SEM. All imaging was performed using the solid state concentric backscatter detector at a working distance of 4 mm.

Since the gold particles can be localized with high precision (better than 5 nm) in both SRM and SEM, they were used to align and register all correlative SRM—SEM datasets. Image registration was done using custom scripts based on the Control Point Registration toolbox in Matlab (Mathworks, Natick, MA). Typically a total number of 15–20 gold nanoparticles were used to register each SRM-SEM dataset.

### Quantitation of cell distortion in correlated SRM and SEM images

**Fig 1:**
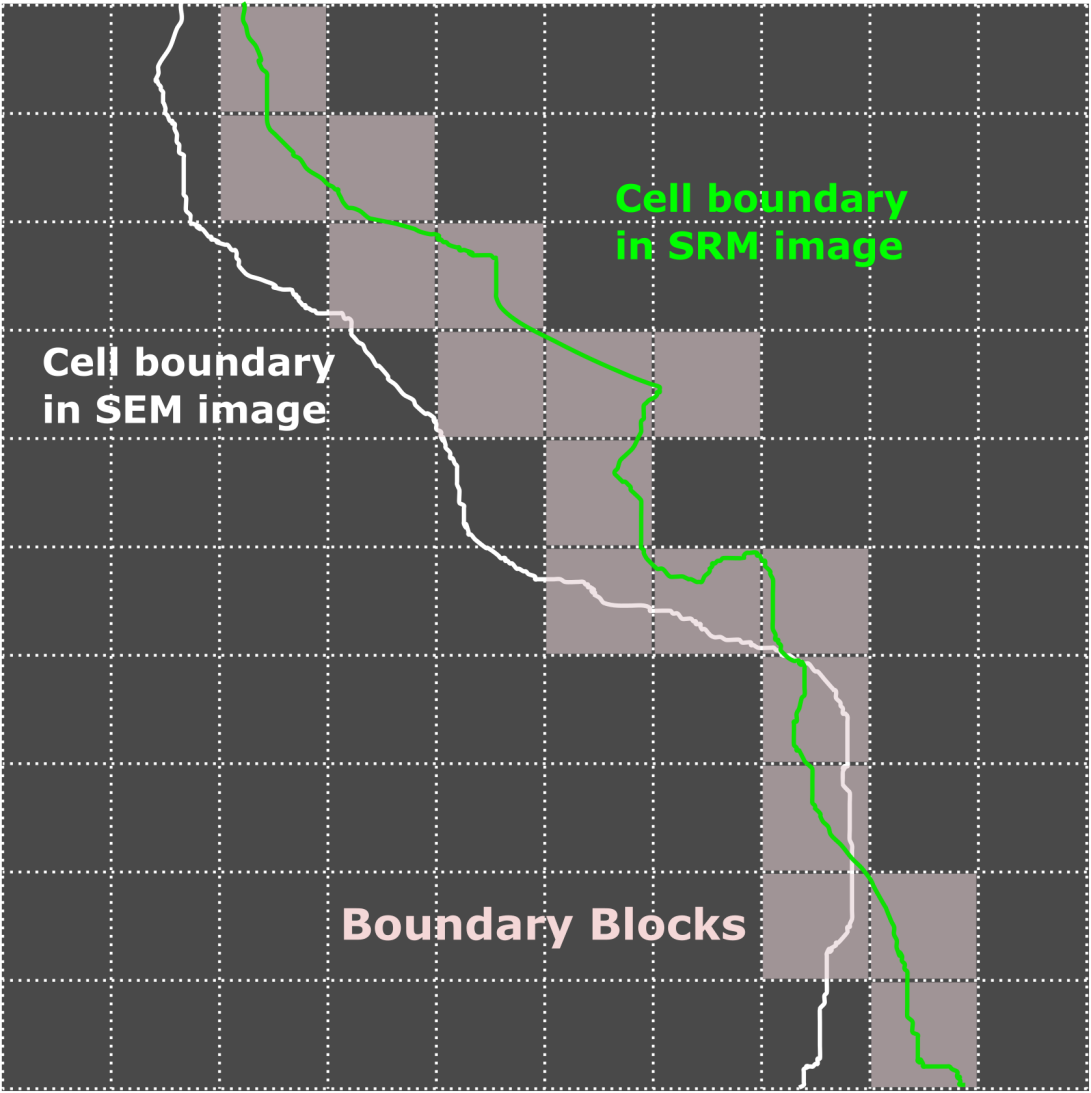
Schematics for quantitating morphological changes in correlative SRM and EM images. After image registration, cell boundaries in both SRM (green) and SEM (white) images are defined, for example with custom Matlab scripts. The SRM image is also divided into 100 x 100 nm^2^ blocks. The difference in cell areas are then calculated and normalized to the contour length determined by counting the number of blocks (gray) along the cell boundary in the SRM image (green). As such, both the area difference and contour length have the units of nm^2^.

After image registration using gold nanoparticles as fiduciary markers, correlated SRM and SEM images were both analyzed with custom Matlab scripts to define the cell boundaries using functions built in the Imaging Processing Toolbox. The distortion in cell morphology was calculated as the total area between the cell boundaries (white and green lines in Fig 1) in the two images, which is dependent on the contour length of the cell boundary. To obtain a normalized distortion value, the SRM image was first divided into 100 x 100 nm^2^ blocks (Fig 1); the total area of boundary blocks (Fig 1, gray) was then determined by counting the number of blocks at the SRM image boundary (Fig 1, green), which is then used to normalize the area difference between the two boundaries. This yields a *Distortion Index* defined as,

$$Distortin\;Index=\frac{Total\;area\;between\;boundaries}{Total\;area\;of\;boundary\;blocks}$$

In essence, the *Distortion Index* describes the average distance between the two boundaries in units of 100 nm; in other words, a *Distortion Index* of 1 indicates that there is a 100 nm distance across the two cell boundaries on average. Of note, both retraction and expansion contribute positively to the total area difference between boundaries.

## Results

### Correlative PALM and SEM imaging

**Fig 2:**
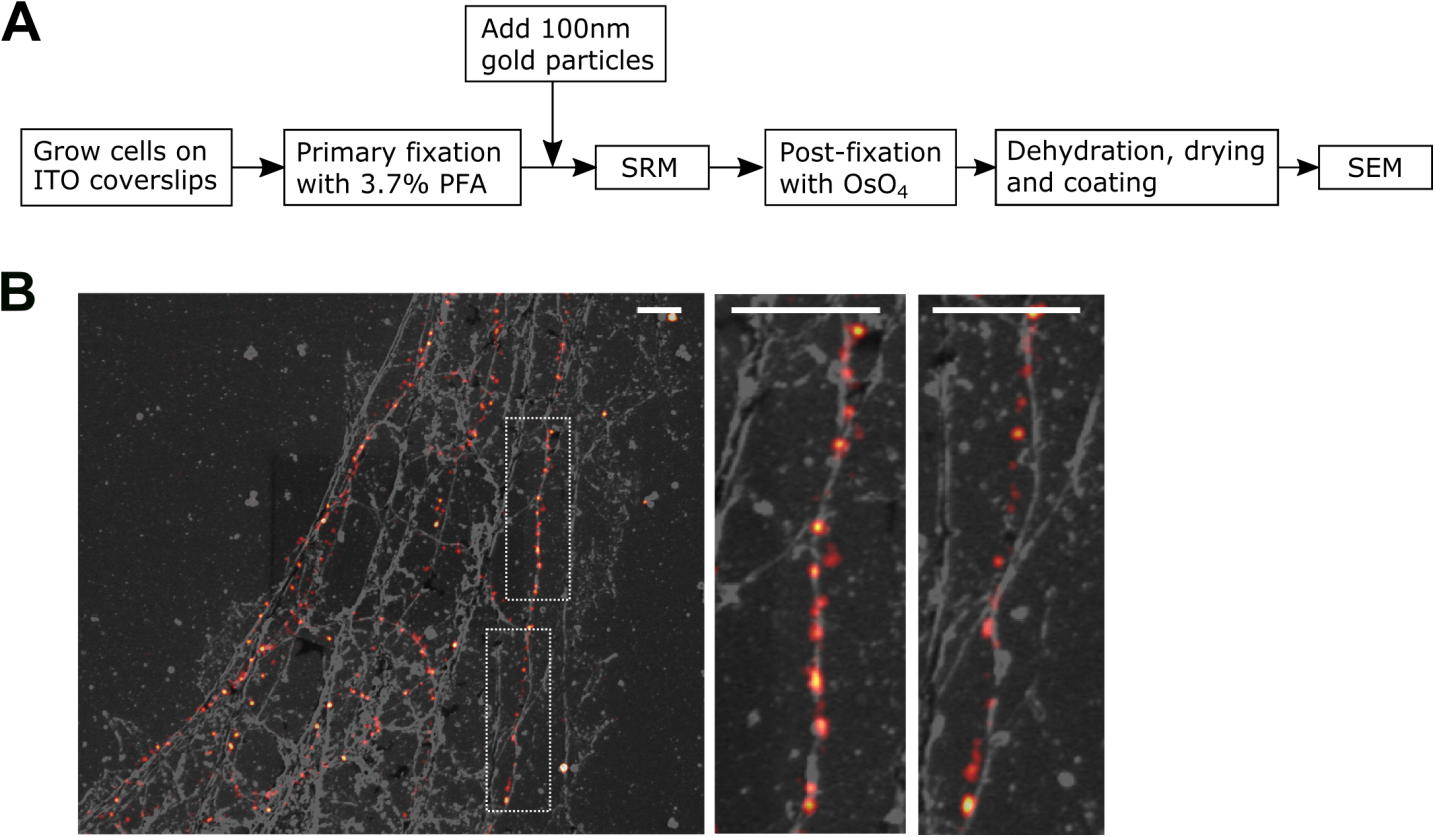
Correlative SRM and SEM imaging. (A) Workflow of imaging the same cell with correlative SRM and SEM; (B) Correlative SRM and SEM image of U2OS cells expressing mEos4 tagged tubulin. Magnified views of the boxed regions in the left image are shown to the right. Scale bars, 1 μm.

We first tested the correlative workflow (Fig 2A) by imaging microtubules. Microtubules are about 25 nm in diameter and are typically single filaments; the microtubule network in U2OS cells was well resolved by imaging mEos4-tubulin with PALM (red in Fig 2B). Here the cells were briefly extracted with detergent to remove tubulin monomers, the membrane, and many other soluble cytosolic components; the cytoskeleton, including microtubules, was left behind in this process. The sample then went through SEM sample preparation and imaging. We were able to identify the same regions that were imaged with PALM during SEM imaging. In SEM images, filamentous structures are also clearly visible, of which a subset represent microtubules (gray in Fig 2B). Indeed, many microtubule filaments revealed by PALM overlap well with filaments in the SEM image (Fig 2B, middle panel). A significant fraction of microtubule filaments, however, overlap loosely between the two images (Fig 2B, right panel), indicating morphological changes during the sample preparation.

### Effects of different drying methods and OsO_4_ post-fixation on cell morphology

**Fig 3:**
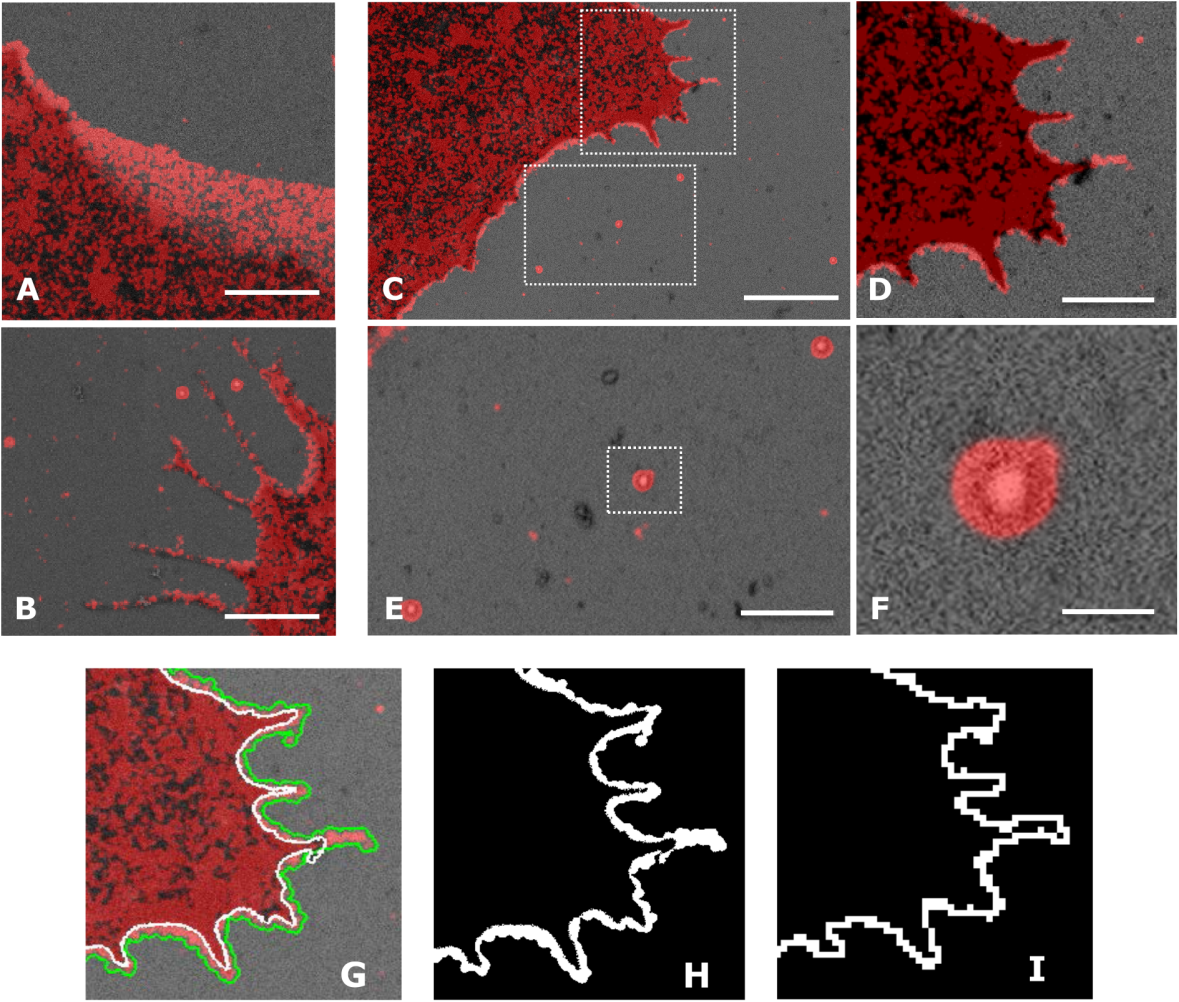
Image registration and quantitation of cell distortion. (A, B, C) Registered SRM and SEM image of U2OS cells expressing PAmCherry1 tagged with the C-terminal tail of HRas. Distortions in cell morphology were evident in multiple regions of the cell; (D, E) Magnified views of the boxed regions in (C); Gold particles are both fluorescent and electron dense and thus were seen in both the SRM and the SEM images (E, F); (F) Magnified view of the gold particles in (E); (G) Defining cell boundaries in the SRM (green line) and the SEM (white line) image; (H) Area difference between the cells boundaries in the two images in (G); (I) Total area of the blocks that fell on the boundary of the SRM image (‘boundary blocks’) were used to represent the contour length in units of nm^2^ and to normalize the area difference. Scale bars, 1 μm in (A, B, D and E), 2 μm in (C), and 100 nm in (F).

In order to quantitate morphological changes during different SEM sample preparation procedures, we applied the workflow shown in Fig 2A to image U2OS cells overexpressing PAmCherry1-tH, which targets to the cell membrane and demarcates the cell boundary with approximately 20 nm spatial resolution in the PALM images (Fig 3A–3G, red). Next, the sample was processed for SEM imaging and the same cell of interest was located (Fig 3A–3G, gray). In both PALM and SEM images, 100 nm gold nanoparticles were present, allowing us to register the two images with high precision. Of note, the SRM images were rendered by using all raw localizations without combining any events even if they were from the same molecules or gold nanoparticles; in this case, the gold nanoparticles appeared as a halo of 80–100 nm, but the mean location of the gold nanoparticle could be determined with better than 5 nm accuracy (typically 2–3 nm). The mean locations of gold nanoparticles in the SRM images were used for registration with the corresponding SEM images to achieve better than 20 nm precision (typically better than 10 nm, Fig 3E and 3F). Differences in the cell boundaries became evident at this resolution in the registered PALM–SEM images (Fig 3G), which were quantitated using the approach described in Fig 1, as shown in Fig 3G–3J.

**Fig 4:**
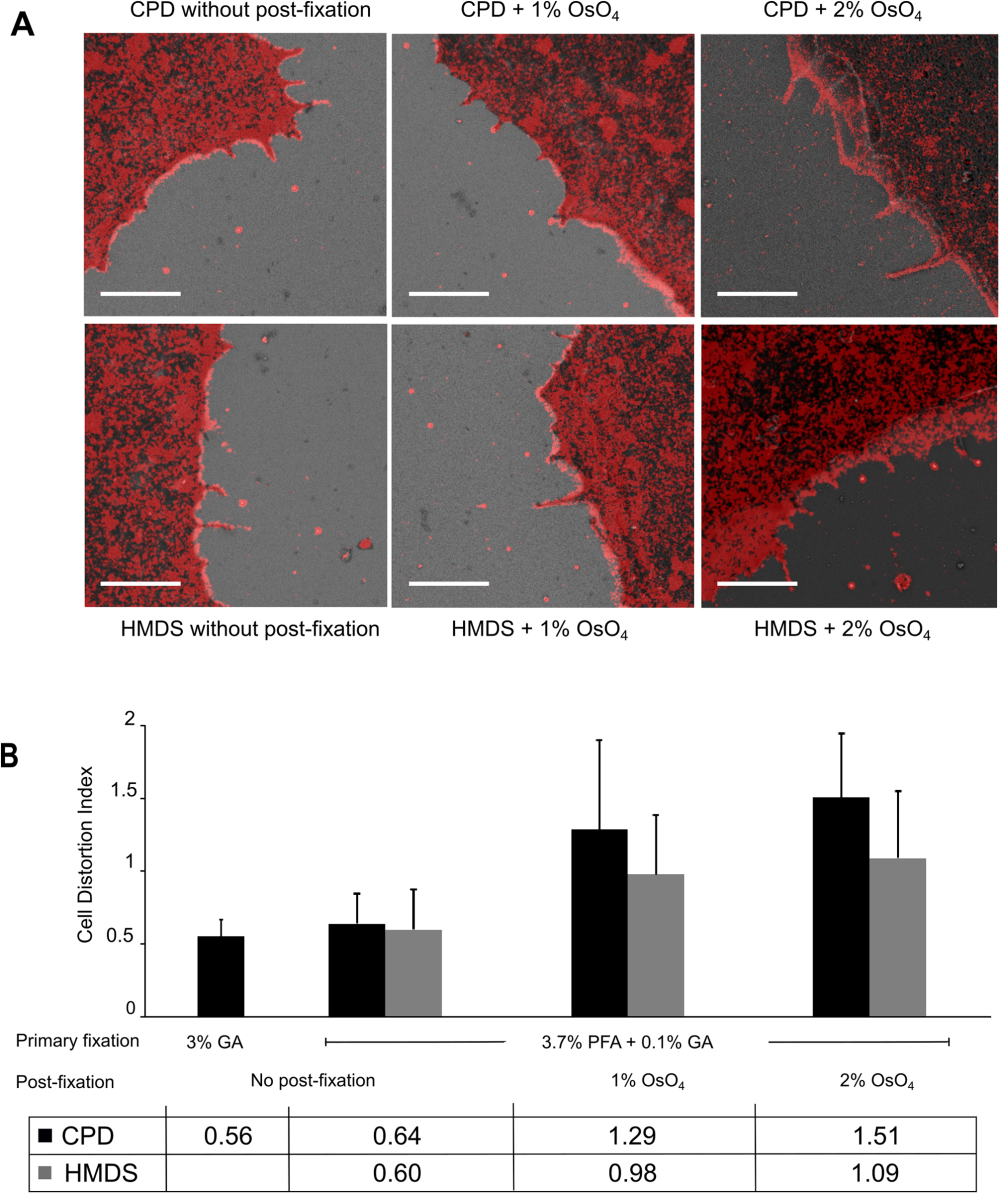
Cell distortion under different fixation and drying conditions. In sample either dried with CPD or HMDS, larger cell distortions were observed with OsO_4_ post-fixation (A). The resulting images were then quantitatively analyzed for distortion (B) as described earlier. Effects of different primary fixation, drying and post-fixation conditions on cell distortion were examined. For each condition, images of 16–20 cells from 4–5 experiments were analyzed. Error bars represent standard deviations. Scale bars, 2 μm in (A).

We have also observed retraction of cell boundaries by ~100 nm, more evidently at the trailing edge (Fig 3A) than at the leading edge, which usually displays a high density of protrusions (Fig 3B). In order to obtain unbiased quantitation, we calculated cell distortion from different regions of the cell boundary that make up the cell contour. Fig 4 shows example images and the quantitation results of cell distortion under different fixation and drying methods. Among all the situations examined, shrinkage accounts for most of the cell distortions. Boundary retraction was apparent in samples dried with either CPD or HMDS. For samples fixed with the 3.7% paraformaldehyde and 0.1% glutaraldehyde only, the two drying methods resulted in a cell distortion index of 0.64 and 0.60, corresponding to an average cell border retraction of 64 and 60 nm, respectively. For samples dried with CPD, primary fixation using 3% glutaraldehyde slightly reduced the distortion index from 0.64 to 0.56, compared with using 3.7% paraformaldehyde and 0.1% glutaraldehyde as the primary fixative (Fig 4B). However, Strong glutaraldehyde fixation could negatively impact the immunogenicity of biological specimen due to its high crosslinking activity [23,24]. Hence, for correlative imaging, 3.7% paraformaldehyde and 0.1% glutaraldehyde is deemed more suitable without sacrificing sample quality for SRM [25].

Additionally, we have examined whether OsO_4_ post-fixation helps to preserve cell morphology and to reduce cellular distortion. After primary fixation and SRM imaging, U2OS cells were post-fixed with 1% or 2% OsO_4_ before dehydration and drying. We found that post-fixation by OsO_4_ caused greater cell distortions compared to the samples only fixed by aldehyde buffer, and the distortions increased with higher OsO_4_ concentration (Fig 4A). Among samples dried by CPD, the calculated cell distortion index with 1% OsO_4_ post-fixation was 1.29, which doubles the index of samples without OsO_4_ post-fixation (0.64). The distortion index further increased to 1.51 in samples treated with 2% OsO_4_. Of note, these distortion index values represent an average magnitude of cell membrane retraction of 64 nm, 128 nm and 151 nm for no OsO_4_ post-fixation, 1% and 2% OsO_4_ post-fixation, respectively. A similar trend was observed for HMDS dried samples, where post-fixation with 1% and 2% OsO_4_ led to evidently more cell distortion.

### Characterizing effects of OsO_4_ post-fixation on the sample with SRM

To further verify effects of OsO_4_ post-fixation on the observed morphological changes, we took STORM images of the same cells before and after OsO_4_ post-fixation. In this case, the sample remained hydrated and no drying or other steps for SEM sample preparation were involved. We labeled the cells with wheat germ agglutinin (WGA) conjugated to Alexa Fluor 647 for STORM imaging. WGA is a carbohydrate-binding protein that recognizes sialic acid and N-acetylglucosaminyl sugar residues, both highly abundant on the plasma membrane[26]. Fluorescent WGA effectively labels plasma membranes and can be used as a counter stain to outline cells in STORM imaging.

**Fig 5:**
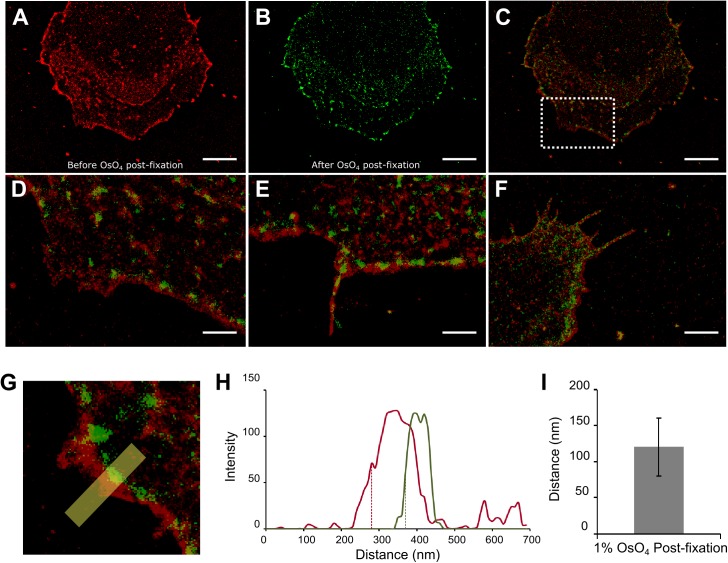
Effects of OsO_4_ post-fixation on cell morphology. (A) STORM image of a cell stained with WGA conjugated to Alexa Fluor 647; (B) STORM image of the same cell after post-fixation with 1% OsO_4_ and re-staining with WGA-AF 647; (C) Overlaid image of (A) and (B); (D) Magnified view of the boxed region in (C); Cell shrinkage (D), distortion of protrusions (E), and discontinued membrane features (F) after OsO_4_ post-fixation were observed; (G-I) Quantitation of morphological changes after OsO_4_ post-fixation. In the absence of a continuous cell boundary after post-fixation, the morphological changes were quantitated by comparing line profiles of short segments at the cell boundary, where features were present in both images (G). In both line profiles, the ‘forefront’ of the cell boundary was defined as the pixel position at the half maximum intensities (H), and the distortion was then calculated as the distance difference between the two positions; (I) Average cell distortion after 1% OsO_4_ post-fixation (*n* = 20). The error bar represents standard deviation. Scale bars, 5 μm in (A, B and C), 1 μm in (D, E) and 2 μm in (F).

STORM images of the same cells before and after post-fixation were registered using the same Matlab routines as described for SRM-SEM image registration (Fig 5A–5C). Changes in cell morphology, including cell shrinkage, protrusion distortion and discontinued membrane features after 1% OsO_4_ post-fixation were commonly observed (Fig 5D–5F). Additionally, much of the WGA labeling was lost after OsO_4_ post-fixation, and the cell boundary in the STORM images became discontinuous, making it difficult to accurately define the cell contour. To quantitate cell boundary distortion, we draw a broad line perpendicular to each visually detected membrane segment in the registered pre- and post-fixation SRM images (yellow line in Fig 5G). Then the intensity profiles of both pre- (red in Fig 5G) and post- (green in Fig 5G) fixation image segments were plotted along the distance of the line (Fig 5H). The cells boundaries from both channels were defined as the position at the half maximum intensities (dashed line in Fig 5H). Cell distortion was then calculated as the distance difference between the two boundary positions. As such, we clearly observed that 1% OsO_4_ post-fixation alone caused an average of 120 nm cell boundary retraction on the sample, even in the absence of dehydration or drying steps. Damage to the WGA signal was too high in samples treated with 2% OsO_4_ to permit a reliable estimation of the morphological change (data not shown).

### Fibronectin coating alleviates morphological changes by OsO_4_ post-fixation

Since less cell distortion was observed mostly at the leading edge of cells, improving cell adhesion may help preserve cell morphology. Among other cell adhesion molecules, fibronectin is an extracellular matrix protein that mediates cellular interactions with extracellular matrix components via integrins and other fibronectin receptors. It is often used to coat glass or plastic surfaces to enhance cell attachment *in vitro*. We therefore investigated whether fibronectin would help stabilize cell morphology during SEM sample preparation.

**Fig 6:**
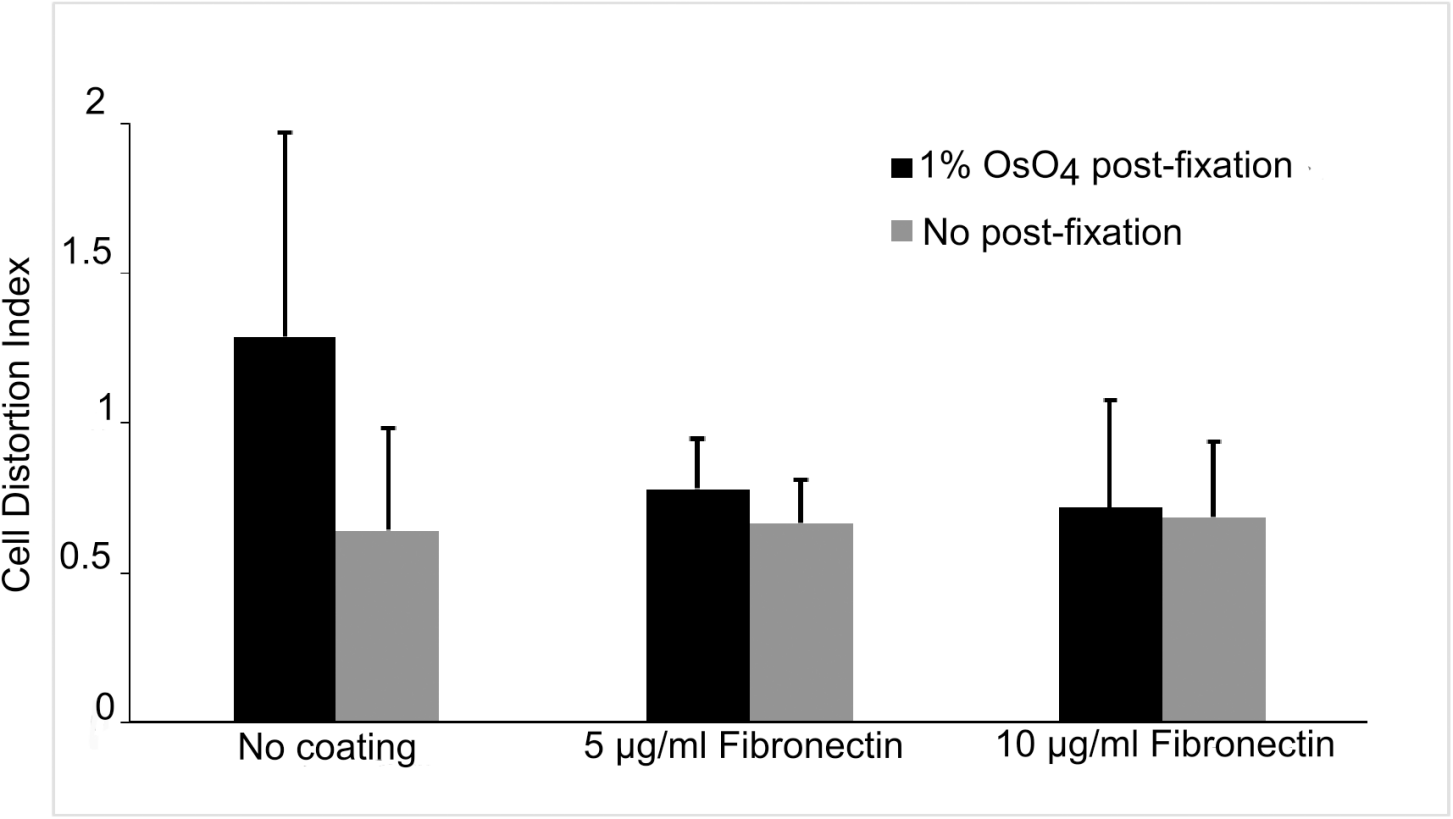
Growing cells on fibronectin-coated coverslips reduced morphological changes by OsO_4_ post-fixation. The cells were grown on non-coated coverslips (left) or those coated with 5 5 μg/mL (middle) or 10 μg/mL (right) fibronectin, fixed in an aldehyde buffer, then processed either directly (gray bars) or after OsO_4_ post-fixation (black bars) for SEM. The cell distortion index was reduced from 1.29 to 0.78 and 0.72 after coating with 5 or 10 μg/mL fibronectin, respectively (black bars). For samples only fixed with the aldehyde buffer (3.7% paraformaldehyde plus 0.1% glutaraldehyde, gray bars), morphology preservation was not evidently improved by fibronectin coating.

Indeed, cells grown on ITO coverslips pre-coated with 5 or 10 μg/mL fibronectin showed much reduced the cellular distortion caused by OsO_4_ post-fixation (Fig 6). Cell distortion indices were reduced from 1.29 to 0.78 and 0.72 with 5 and 10 μg/mL fibronectin coating, respectively. With fibronectin coating, the distortion indices were approaching that of cells grown on bare ITO without OsO_4_ post-fixation (0.64). Importantly, fibronectin coating did not improve morphological distortion index for samples that were fixed only with primary aldehyde fixatives (Fig 6). These data suggest that substrate coating with fibronectin mostly protected cells from distortion by OsO_4_ post-fixation but not from shrinkage during dehydration and drying.

## Discussion

Correlative SRM and EM promises to be a powerful tool for studying protein localization and organization in the native cellular context [27]. For workflows that perform SRM before EM, it is important to ensure that the cell morphology is well preserved when the sample goes through the preparation steps. In this study, we have examined morphological changes to adherent cells grown on a solid substrate during SEM sample preparation, by imaging the cells with SRM in hydrated state prior to SEM sample processing. Our results indicate that the degree of cell distortion, measured as the differences between the cell boundaries defined by SRM and SEM images, ranges from ~60 nm to ~130 nm depending on the sample processing protocol. For example, post-fixation with OsO_4_ causes more pronounced cell boundary retraction whereas fibronectin coating helps reduce the morphological change. These quantitative measurements help to identify factors that contribute to cell distortion during SEM sample processing and to develop strategies to better preserve cellular structure.

OsO_4_ has been extensively used as a post fixative and staining reagent in EM for preserving ultrastructure and providing membrane contrast, but its exact effects on biological specimen have not been clearly understood. Our study shows that OsO_4_ causes slight shrinkage of the cell boundaries. This shrinkage is likely due to the strong oxidizing activities of OsO_4_ that, while important for staining membranes [28], may also destroy some adhesion molecules that help the cell to attach to the substrate. Consistently, we have observed that OsO_4_ post-fixation caused loss of WGA-binding sites in cells, some of which is attributed to cell surface glycans. This adverse effect of OsO_4_ is further corroborated by the fact that coating the substrate with fibronectin almost completely reversed the shrinkage caused by OsO_4_ treatment. Here, the role of fibronectin is likely to enhance cell attachment to the substrate, by making the cell—substrate interface more resistant to OsO_4_ treatment.

Similar effects of cell attachment on OsO_4_ resistance were also observed on cells with a leading and trailing edge, indicative of cell migration. As shown in Fig 3A and 3B, the leading edge of the cell usually experiences significantly less morphological changes than the trailing edge. The leading edge is typically characterized by a flat morphology and a higher density of protrusions, where integrins, extracellular matrix proteins, and adapter proteins form adhesion complexes to mediate cell attachment[29]. The trailing edge is where the adhesion complexes dissolve to allow the cell body to migrate in the direction of movement, resulting in a loose attachment with the substrate. Hence, coating with fibronectin and potentially other molecules that improve cell adhesion seems a viable approach to counter the cell boundary retraction observed during OsO_4_ post-fixation.

It is worth mentioning that previous reports seem to mostly suggest that OsO_4_ post-fixation cause cell swelling, which was attributed to a difference between the osmotic pressure of the fixative and that of the cytoplasm[12]. This does not necessarily contradict our observation, because a retraction in cell boundary does not prevent the rest of the cell body from expanding. For example, the cells may have a slightly reduced footprint but become significantly taller (i.e., overall rounding of the cell), and the net result is increased volume. Therefore, a complete assessment in the morphological change of cells during SEM sample preparation should compare SRM and SEM images of the same cell in all three dimensions. While whole cell imaging with SEM is possible with volumetric techniques such as FIB-SEM, similar imaging with SRM with comparable resolutions is still in development[30]. Future work can utilize three dimensional super-resolution techniques combined with volumetric SEM (such as FIB-SEM) to evaluate these effects during sample preparation.

In this study, we also compared CPD and chemical drying with HMDS, which are two of the most commonly used drying methods for biological specimen. Our results indicate that CPD and HMDS yielded comparable cell boundary distortion. Between the two, CPD has been the popular drying method designed to avoid artifacts due to surface tension. HMDS is a cheaper and time-saving alternative that has been proven to work with most biological sample types [3,31,32]. Together, CPD may still offer some advantages in terms of preserving cell morphology although HMDS seems to suffice for many samples.

Approaches for correlative SRM and EM have been reported for imaging plastic-embedded TEM samples. The sample preparation procedures for those samples including high pressure freezing, freeze substitution and resin embedding are critical for preserving cell morphology and cellular structures. Preserving cell morphology in these experiments is similarly of concern, and the workflows developed here could potentially be adopted for quantitation of morphological changes in those cases. With information provided through these measurements, specific steps in the sample processing procedures of these experiments may be modified for optimal preservation of specimen morphology and ultrastructure.
